# Medullary thyroid cancer in a 9-week-old infant with familial MEN 2B: Implications for timing of prophylactic thyroidectomy

**DOI:** 10.1186/1687-9856-2012-25

**Published:** 2012-09-19

**Authors:** Roopa Kanakatti Shankar, Michael J Rutter, Steven D Chernausek, Paul J Samuels, Jun Qin Mo, Meilan M Rutter

**Affiliations:** 1 Division of Endocrinology, Cincinnati Children’s Hospital Medical Center, MLC 7012, 3333 Burnet Avenue, Cincinnati, OH, 45229, USA; 2 Division of Otolaryngology, Cincinnati Children’s Hospital Medical Center, MLC 2018, 3333 Burnet Avenue, Cincinnati, OH, 45229, USA; 3 Department of Pediatrics, University of Oklahoma Health Sciences Center, 1200 N Phillips Ave, Suite 4500, Oklahoma City, OK, 73104, USA; 4 Department of Pediatric Anesthesiology, Cincinnati Children’s Hospital Medical Center, MLC 2001, 3333 Burnet Avenue, Cincinnati, OH, 45229, USA; 5 Division of Pathology, Cincinnati Children’s Hospital Medical Center, MLC 1010, 3333 Burnet Avenue, Cincinnati, OH, 45229, USA

**Keywords:** Medullary thyroid cancer (MTC), Multiple endocrine neoplasia, Infant, Hereditary cancer, Thyroidectomy

## Abstract

**Background:**

Patients with Multiple Endocrine Neoplasia type 2 (MEN 2) are at high risk of developing aggressive medullary thyroid carcinoma (MTC) in childhood, with the highest risk in those with MEN type 2B (of whom >95% have an M918T RET proto-oncogene mutation). Metastatic MTC has been reported as young as 3 months of age. Current guidelines recommend prophylactic thyroidectomy within the first year of life for MEN 2B.

**Patient findings:**

We report a 9-week-old infant with MTC due to familial MEN 2B. A full-term male infant, born to a mother with known MEN 2B and metastatic MTC, had an M918T RET proto-oncogene mutation confirmed at 4 weeks of age. He underwent prophylactic total thyroidectomy at 9 weeks of age. Pathology showed a focal calcitonin-positive nodule (2.5 mm), consistent with microscopic MTC.

**Summary:**

This case highlights the importance of early prophylactic thyroidectomy in MEN 2B. Although current guidelines recommend surgery up to a year of life, MTC may occur in the first few weeks of life, raising the question of how early we should intervene. In this report, we discuss the risks, benefits and barriers to performing earlier thyroidectomy, soon after the first month of life, and make suggestions to facilitate timely intervention. Prenatal anticipatory surgical scheduling could be considered in familial MEN 2B. Multidisciplinary collaboration between adult and pediatric specialists is key to the optimal management of the infant at risk.

## Background

Multiple Endocrine Neoplasia type 2 (MEN 2) is an autosomal dominant hereditary cancer syndrome with a prevalence of about 1 in 30,000 [1]. Germline mutations in the RET (Rearranged during Transfection) proto-oncogene account for the three distinct clinical phenotypes, MEN 2A, MEN 2B and familial medullary thyroid cancer (MTC). All the MEN 2 syndromes are associated with MTC, which frequently metastasizes to regional and distant sites, and is the primary MEN-related cause of death in affected patients. Thus, prophylactic thyroidectomy is recommended in asymptomatic patients with pathogenic mutations, before the development of MTC. Strong genotype-phenotype correlations have enabled risk stratification and form the basis of recommendations for timing of prophylactic surgery [2].

MEN 2B is the rarest and most aggressive form of MEN 2, and is associated with early-onset MTC, as well as pheochromocytoma, mucosal neuromas, intestinal ganglioneuromas and a Marfanoid habitus. More than 95% of cases with MEN 2B have an M918T mutation in exon 16 of the RET proto-oncogene; a further 2-3% have an A883F mutation [3]. Both mutations are classified as level 1 or ATA-D, the highest risk category for aggressive MTC, according to the current ATA (American Thyroid Association) guidelines [1]. These guidelines state that "infants with ATA-D mutations (MEN 2B) should undergo prophylactic thyroidectomy as soon as possible and within the first year of life in an experienced tertiary care setting". Former consensus guidelines had recommended thyroidectomy "within the first six months and preferably within the first month of life" [2].

We describe one of the youngest patients with biopsy-proven MTC at 9 weeks of age associated with familial MEN 2B. The manifestation of MTC at this young age underscores the importance of early diagnosis and thyroidectomy. Although current guidelines allow for surgery up to a year of age, MTC may occur much earlier in infancy, raising the question of how soon intervention should optimally occur. We make simple and practical suggestions to achieve an earlier thyroidectomy, after the first month of life, in these high-risk individuals.

## Case presentation

A male infant was born to a mother with known MEN 2B due to an M918T RET proto-oncogene mutation. His mother presented at 13 years of age with mucosal neuromas, a typical Marfanoid habitus and MTC. She underwent total thyroidectomy and lymph node dissection. Histopathology confirmed multifocal MTC with multiple lymph node metastases. She later developed hepatic and pulmonary metastases, and underwent resection of a pheochromocytoma at age 25 years. She is currently alive and well at 27 years of age, but with persistent MTC and a serum calcitonin concentration over 2000 pg/mL.

**Figure 1:**
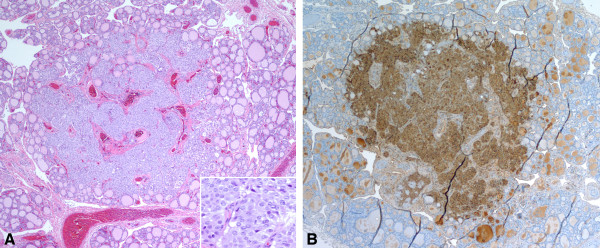
** A thyroidectomy specimen of the patient reveals a 2.5 mm, poorly defined tumor nodule with focal infiltrative borders.** The tumor cells are relatively uniform with moderately eosinophilic cytoplasm, and round to slightly irregular nuclei with open chromatin (1**A** and inset). The tumor cells show strong reactivity to calcitonin immunohistochemical staining (1**B**).

The infant was delivered at term with a birth weight of 3.26 kg, and had no symptoms or signs of MEN 2B. He underwent genetic testing at 4 weeks of age, which confirmed the same mutation. He was then referred to Pediatric Otolaryngology and Endocrinology. His circulating calcitonin concentration at 4 weeks was 81 pg/mL (41 pg/mL is the 95th percentile for age less than 6 months). He underwent prophylactic thyroidectomy at 9 weeks of age. Parathyroid glands were visualized and left in situ. There were no visible or palpable lymph nodes at the time of surgery. His perioperative course was unremarkable, and he recovered without complication. Histopathology of the thyroid showed a 2.5 mm nodular lesion with infiltrative borders (Figure 1A) and cells which were reactive for calcitonin on immunohistochemical staining (Figure 1B), consistent with microscopic MTC. There was no vascular invasion.

Post-operatively, his serum calcitonin concentration declined to 8 pg/mL, and has remained in this range since. At 2 years of age, his carcinoembryonic antigen concentration is 2.1 ng/mL (normal <5 ng/mL) and thyroglobulin is undetectable (consistent with no residual thyroid tissue). He has no residual thyroid tissue or lymph node enlargement by ultrasound examination. He has normal growth and development, but has chronic constipation due to biopsy-proven intestinal ganglioneuromatosis. He has absent tearing. He has developed a tongue nodule, likely a mucosal neuroma.

## Discussion

We report one of the youngest patients in the literature with MTC due to familial MEN 2B at 9 weeks of age. Histologically, the tumor was 2.5 mm in size and had infiltrating borders in focal areas, confirming the diagnosis of microscopic MTC and distinguishing it from C-cell hyperplasia [4]. Other reports of early MTC in MEN 2B include a microscopic MTC in a 9-week-old infant, but with a de novo mutation [5], and metastatic MTC in a 3-month-old infant with familial MEN 2B [6].

Although extensive long term follow-up data is lacking in MEN 2B [7], the benefit of prophylactic thyroidectomy before the onset of metastatic MTC is well accepted [1]. The current guidelines state that prophylactic thyroidectomy for ATA-D (highest risk) mutations must be undertaken "as soon as possible and within the first year of life" [1]. Our case, together with these other reports, highlights the importance of early prophylactic surgery during infancy. However, while the guidelines allow for surgery up to a year of age, our case raises the question as to whether intervention should occur “as soon as possible” in earlier infancy. In familial cases, when the diagnosis is anticipated, thyroidectomy soon after the first month of life could be considered.

Current hurdles to performing prophylactic total thyroidectomy in the first few months of life include: 1) time delays in arriving at a diagnosis and scheduling surgery, 2) concern that surgical and anesthetic risks outweigh the benefits of surgery at that age, and 3) concern for iatrogenic hypothyroidism.

Any impediment to the multiple steps associated with the treatment of these patients, including genetic testing, referral to a pediatric thyroid surgeon, pre-surgical evaluation and procedure scheduling, could result in significant surgical delay. An early diagnosis should be made by genetic testing of at risk infants soon after birth, or even prenatally. The ATA guidelines recommend that all patients with MEN 2B of child-bearing age should be offered prenatal genetic counseling [1]. This includes discussion of the options of pre-implantation genetic testing (enabling implantation of unaffected embryos using assisted reproductive techniques), chorionic villus sampling, and amniocentesis. Prenatal genetic counseling helps parents understand the risk of having an affected infant (50%) and the importance of prophylactic thyroidectomy in an asymptomatic baby, and facilitates RET testing early in the newborn period. In addition, if surgical referral and scheduling of anticipated thyroidectomy (contingent upon subsequent confirmation of the diagnosis) occur prenatally in familial cases, postnatal genetic confirmation may become the sole rate-limiting step. Of note, while pre-operative calcitonin measurement is recommended, calcitonin concentrations should be interpreted with caution in infants below 6 months of age and should not be used to dictate surgical decisions [1].

There are potential surgical and anesthetic risks associated with performing thyroidectomy during early infancy. Children younger than six years of age have the highest rate of complications following thyroidectomy as compared with adults, although outcomes may be improved by referral to high-volume surgeons [8]. Significantly delaying prophylactic thyroidectomy may improve patient safety, but is not an option in MEN 2B when surgery has to be undertaken by a year of age, and possibly sooner. There are no formal studies of thyroidectomy outcomes in infants below a year of age. However, it is our opinion that there is little additional surgical risk for an infant one month of age compared with six months of age when performed by an experienced pediatric thyroid surgeon working in an environment with appropriate pediatric resources. Surgical complications include hypoparathyroidism and recurrent laryngeal nerve injury. Post-operative hypoparathyroidism does not appear to be related to the age of surgery in children with MEN [9], while the risk of both complications is increased by lymph node dissection [10]. An added benefit of early surgery soon after a month of age in familial MEN 2B is that central neck dissection may be avoided. According to the ATA guidelines, “thyroidectomy prior to lymph node metastases obviates the need for central compartment lymph node dissection which is associated with a higher rate of hypoparathyroidism and vocal cord paralysis” [1].

There is evidence that the risk of general anesthesia is greater in a child under one year of age [11]. However, anesthetic risk can be attenuated by carefully screening for children with a history of prematurity, congenital heart disease, or other congenital abnormalities [12]. We typically wait until a full-term infant is one month of age before administering anesthesia for an elective procedure in order to minimize the risk of post-operative apnea. We therefore suggest that thyroid surgery be undertaken after 30 days of age in a child with MEN 2B.

Finally, the consequences of undertreating iatrogenic hypothyroidism are a potential problem. This concern should not warrant delaying thyroidectomy; instead, intensive monitoring and careful replacement of levothyroxine is essential [13].

## Conclusions

**Figure 2:**
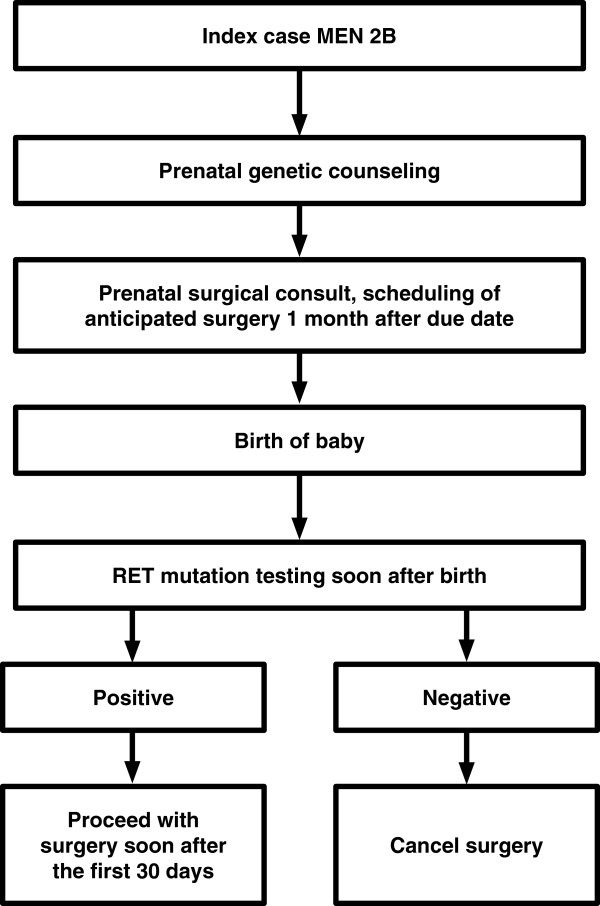
Suggested algorithm to facilitate early thyroidectomy during infancy.

Our case highlights the importance of early prophylactic thyroidectomy, and raises consideration of performing surgery “as soon as possible” after the first month of life in familial MEN 2B, without waiting up to a year of age. After considering risks and benefits, we propose a simple and practical algorithm to facilitate timely prophylactic thyroidectomy in early infancy in familial MEN 2B (Figure 2). Families should receive prenatal genetic counseling according to ATA guidelines [1]. In addition, we suggest prenatal referral to a surgeon with expertise in pediatric thyroid surgery, with scheduling of anticipated surgery. After birth, if RET testing is positive, surgery may be performed soon after a month of age. This approach would eliminate time lost in surgical evaluation and scheduling after genetic results are known. Ideally, surgery should be undertaken at a tertiary care center by an experienced pediatric thyroid surgeon. In addition, the participation of a pediatric anesthesiologist and access to skilled post-operative pediatric nursing would minimize the risk of peri-operative complications.

While prophylactic thyroidectomy in inherited MEN 2B is a unique pediatric problem, the gatekeepers to appropriate referral and timely intervention are frequently adult endocrinologists and obstetricians. Early collaboration between adult and pediatric specialists is key to optimizing the management of the infant with familial MEN 2B.

## Consent

Written informed consent was obtained from the patient’s mother for publication of this case report and any accompanying images. A copy of the written consent is available for review by the Editor-in-Chief of this journal.

## Competing interests

The authors declare that they have no competing interests.

## Authors’ contributions

All authors have made substantive intellectual contributions to this paper. All authors have contributed to the concept and design of the case report. RKS, MJR, SDC and MMR have diagnosed and/or treated the patient and/or mother. JQM was the pathologist for the case. RKS, MJR and MMR drafted the manuscript, and all authors have critically revised the manuscript for important intellectual content. All authors have given final approval of the version to be published.

## References

[B1] Kloos RT Eng C Evans DB Francis GL Gagel RF Gharib H Moley JF Pacini F Ringel MD Schlumberger M Wells SA Medullary thyroid cancer: management guidelines of the American Thyroid Association Thyroid 2009 19 565 612 10.1089/thy.2008.0403 19469690

[B2] Brandi ML Gagel RF Angeli A Bilezikian JP Beck-Peccoz P Bordi C Conte-Devolx B Falchetti A Gheri RG Libroia A Guidelines for diagnosis and therapy of MEN type 1 and type 2 J Clin Endocrinol Metab 2001 86 5658 5671 10.1210/jc.86.12.5658 11739416

[B3] Eng C Clayton D Schuffenecker I Lenoir G Cote G Gagel RF van Amstel HK Lips CJ Nishisho I Takai SI The relationship between specific RET proto-oncogene mutations and disease phenotype in multiple endocrine neoplasia type 2. International RET mutation consortium analysis JAMA 1996 276 1575 1579 10.1001/jama.1996.03540190047028 8918855

[B4] Ashworth M The pathology of preclinical medullary thyroid carcinoma Endocr Pathol 2004 15 227 231 10.1385/EP:15:3:227 15640548

[B5] Unruh A Fitze G Janig U Bielack S Lochbuhler H Coerdt W Medullary thyroid carcinoma in a 2-month-old male with multiple endocrine neoplasia 2B and symptoms of pseudo-Hirschsprung disease: a case report J Pediatr Surg 2007 42 1623 1626 10.1016/j.jpedsurg.2007.05.015 17848262

[B6] Zenaty D Aigrain Y Peuchmaur M Philippe-Chomette P Baumann C Cornelis F Hugot JP Chevenne D Barbu V Guillausseau PJ Medullary thyroid carcinoma identified within the first year of life in children with hereditary multiple endocrine neoplasia type 2A (codon 634) and 2B Eur J Endocrinol 2009 160 807 813 10.1530/EJE-08-0854 19240193

[B7] Waguespack SG Rich TA Multiple endocrine neoplasia [corrected] syndrome type 2B in early childhood: long-term benefit of prophylactic thyroidectomy Cancer 2010 116 2284 20166211 10.1002/cncr.24941

[B8] Sosa JA Tuggle CT Wang TS Thomas DC Boudourakis L Rivkees S Roman SA Clinical and economic outcomes of thyroid and parathyroid surgery in children J Clin Endocrinol Metab 2008 93 3058 3065 10.1210/jc.2008-0660 18522977

[B9] Iler MA King DR Ginn-Pease ME O'Dorisio TM Sotos JF Multiple endocrine neoplasia type 2A: a 25-year review J Pediatr Surg 1999 34 92 96 discussion 96-97 10.1016/S0022-3468(99)90236-1 10022151

[B10] Piolat C Dyon JF Sturm N Pinson S Bost M Jouk PS Plantaz D Chabre O Very early prophylactic thyroid surgery for infants with a mutation of the RET proto-oncogene at codon 634: evaluation of the implementation of international guidelines for MEN type 2 in a single centre Clin Endocrinol (Oxf) 2006 65 118 124 10.1111/j.1365-2265.2006.02559.x 16817830

[B11] Mason LJ An update on the etiology and prevention of anesthesia-related cardiac arrest in children Paediatr Anaesth 2004 14 412 416 10.1111/j.1460-9592.2004.01341.x 15086854

[B12] Bhananker SM Ramamoorthy C Geiduschek JM Posner KL Domino KB Haberkern CM Campos JS Morray JP Anesthesia-related cardiac arrest in children: update from the Pediatric Perioperative Cardiac Arrest Registry Anesth Analg 2007 105 344 350 10.1213/01.ane.0000268712.00756.dd 17646488

[B13] Frank-Raue K Buhr H Dralle H Klar E Senninger N Weber T Rondot S Hoppner W Raue F Long-term outcome in 46 gene carriers of hereditary medullary thyroid carcinoma after prophylactic thyroidectomy: impact of individual RET genotype Eur J Endocrinol 2006 155 229 236 10.1530/eje.1.02216 16868135

